# Gait Classification Based on Micro-Doppler Effect

**DOI:** 10.3390/s26082390

**Published:** 2026-04-13

**Authors:** Yong Chen, Sicheng Li, Chao Qin, Kun Liang, Zuxiang Wei, Hang Zhang

**Affiliations:** School of Automation, Central South University, Changsha 410083, China; chenyong@csu.edu.cn (Y.C.); 8207231503@csu.edu.cn (S.L.); 224143@csu.edu.cn (C.Q.); 8207230275@csu.edu.cn (K.L.); 244612183@csu.edu.cn (Z.W.)

**Keywords:** micro-Doppler effect, zero-phase component analysis Whitening (ZCA Whitening), state-space method (SSM), frequency-modulated continuous wave (FMCW) radar

## Abstract

In this paper, an improved state-space method (SSM) is proposed for gait feature extraction. By introducing zero-phase component analysis Whitening (ZCA Whitening) and an algorithm to search estimated echo as the preprocessing method, pedestrian echoes are divided into three groups according to the frequency probability density: torso, feet, and other segments. Two channels of echoes are selected as inputs to the SSM, which is employed to identify the corresponding micro-Doppler trajectory. On this basis, five gait features of torso amplitude, stride length, walking cycle, torso maximum speed, and feet maximum speed are extracted. Simulation based on the Boulic model, compared with the traditional SSM, demonstrated that there is no need to estimate the model order and that a more accurate torso micro-Doppler trajectory and effective micro-motion features of the feet can be obtained by the proposed method. Finally, 77 GHz FMCW radar was used to collect the echoes of four pedestrians. The classifier was designed based on a support vector machine (SVM), and the classification experiment verified the effectiveness of the extracted gait features.

## 1. Introduction

Because of the unique advantages of radar compared to other sensors, it has been widely used in military and civilian fields [1,2,3,4,5]. The advantages of radar systems are mainly reflected in two aspects: Firstly, radar has long-distance detection capabilities, which can overcome the difficulty of being unable to detect at close distances due to confidentiality, safety, or potential hazards. Secondly, radar can all day and in all weather conditions, making it capable of solving problems associated with the inability of optical equipment to detect and identify objects at night, as well as the inability of infrared equipment to work in places with high heat, such as the scene of a fire.

With the development of science and technology, the target information carried by radar electromagnetic (EM) waves has also become richer, giving birth to the concept of the micro-Doppler effect. Victor C. Chen of the U.S. Naval Research Laboratory first gave the definition of micro-motion: the vibration, rotation, and acceleration of the target or the components of the target other than the translational movement of the center of mass [6], such as the rotation of the rotor, the rotation of jet engine blades, the vibration of a bridge, etc. The micro-motion of a target or target component will produce frequency modulation in the radar echo signal; that is, in addition to the main Doppler frequency shift, there are additional Doppler side bands about the Doppler frequency. This modulation caused by vibration and rotation is called the micro-Doppler effect [7,8].

When a human target is walking, in addition to the movement of the torso (center of mass) modulating the EM signal, the periodic swing of the limbs relative to the center of mass of the human body will also have a nonlinear modulation effect on the EM signal. This nonlinear modulation makes the echo carry rich feature information, such as pedestrian torso size, stride length, stride frequency, etc. It provides a basis for target classification and recognition. Gait recognition is a method of identifying a target through the way a target walks. It is a relatively new biometric identification technology that more and more researchers have paid attention to in recent years. As an emerging biometric technology, it can make up for the shortcomings of some traditional biometric technology. For example, fingerprint recognition requires direct contact, and facial recognition has weak anti-occlusion capabilities. Gait recognition based on micro-Doppler information has important research value in both military and civilian fields [9,10,11,12,13].

Saho et al. and Yang et al. focused on health monitoring and disease-related gait analysis [9,10]. Saho et al. used micro-Doppler radar to classify gait patterns for fall risk assessment in the elderly [9]. Yang et al. detected subtle gait alterations caused by neurodegenerative diseases for early screening [10]. These studies highlight the key advantages of gait recognition, a contactless, sensor-free method suitable for long-term monitoring without cooperation, overcoming the limitations of wearable devices.

Yang et al. tackled disguised gait recognition in open environments for security surveillance. A gait is hard to disguise, and radar works under varying illumination, occlusion, and weather conditions, enabling all-weather, non-cooperative identity verification [11].

To demonstrate lightweight gait analysis on embedded devices, Kim et al. proposed a low-complexity 1D phase modulation scheme [12]. Ma et al. designed a lightweight feature fusion and broad learning framework [13]. These methods make gait recognition feasible for resource-constrained IoT edge devices, expanding applications in smart homes and wearables.

Many researchers have achieved significant results in gait recognition with respect to the micro-Doppler effect in recent years. In [14], Addabbo and Bernardi used low-power FMCW radar to measure and produce a pedestrian echo dataset. Then, a deep spatial–temporal convolutional neural network was applied to classify five targets, with a highest accuracy rate of 94.9%. The authors of [15] collected 77 GHz FMCW radar data of pedestrians walking outdoors on a real urban pedestrian street. The scenes covered pedestrian groups with one to eight people, which were divided into four categories (empty street, one to two people, three to four people, and five to eight people). They employed a small amount of measured data, combined with synthetic data pre-training (transfer learning), to train a convolutional neural network (CNN) for pedestrian group counting and classification experiments. The average classification accuracy reached 70% on measured data alone and was improved to 77% after introducing transfer learning. The authors of [16] proposed a new method of initializing a recurrent neural network based on parallel channel recognition (PC-IRNN). Based on the Boulic model, they simulated a dataset of three types of gait—fast walking, slow walking, and normal walking—and verified the superiority of PC-IRNN by comparing it with a long short-term memory (LSTM) network and a recognition initialization recurrent neural network (IRNN). The classification accuracy rate reached 99.13%.

It is not difficult to see from the above that deep learning is an effective method to realize radar gait recognition. The general process can be summarized as follows:

- Apply short-time Fourier transform (STFT) or another time-frequency analysis method to convert a radar echo into a picture form that is directly or indirectly used as a dataset.
- Complete model training using these picture sets to realize gait recognition using the model.

Although many published results have proven the effectiveness of combining deep learning and the time-frequency analysis method to realize gait recognition, it should be pointed out that, compared with physical gait features such as stride frequency, stride length, and walking speed, the interpretability of the high-order features that a deep learning network extracts is poor, and it is difficult to conduct further research on the basis of such features. In addition, compared with the visual gait dataset, there are few public datasets in the radar field, which directly adversely affects the performance of the deep learning model, which depends on the number of training samples.

Therefore, how to effectively extract physical gait features to achieve high classification accuracy in a small dataset has also been a research hotspot in the field of micro-Doppler in recent years. In [17], a 10.525 GHz coherent CW radar system was developed to measure human micro-Doppler signatures. The authors extracted a set of human gait features using STFT and the Chirplet. After comparison, they found that STFT can provide a better description of human bio-mechanical motion than Chirplet. In addition to STFT, many other time-frequency analysis methods are also used to extract micro-Doppler features that can provide valuable information on human motion dynamics. The authors of [18] performed a second STFT after fast Fourier transformationto obtain a cadence velocity diagram; then, four classification features, including step frequency and spectrum shape, were extracted. The average classification accuracy rate was above 93%. Based on FMCW radar, the authors of [19] collected the radar echoes of human subjects standing still, swinging their arms slowly and vertically, swinging their arms rapidly and vertically, and walking. Then, they proposed a distance-Doppler filter algorithm to improve the observability of human micro-movements in the frequency spectrum, effectively suppressing undesired target responses. In addition to STFT, some other time-frequency analysis methods are also widely used in the extraction of human gait features, such as S transform [20], pseudo-smooth Wigner–Ville distribution [21], Hilbert Chuang transformation [22], etc. It should be pointed out that because pedestrian micro-motion is complicated, pedestrian time-frequency images obtained by traditional time-frequency analysis methods often face the problem of serious multi-component aliasing, and the accuracy of feature extraction is affected by the performance of micro-motion observation in the time-frequency image.

In the case of describing and analyzing a target containing multiple micro-motion components, the separation of different micro-Doppler trajectories helps to make better judgments on the micro-motion form to achieve identification and classification of targets. Therefore, in terms of human gait recognition, in order to obtain detailed information about human movement and extract accurate gait features, it is necessary to identify the trajectory of specific limbs and body joints and distinguish their corresponding micro-Doppler parts in spectrograms. Fortunately, many scholars have done a lot of research on algorithms for the separation of micro-Doppler and have achieved fruitful results. A method based on the Boulic model to estimate the parameters of human walking was proposed in [23], but only simulations were provided, with no experimental results that can be used to verify its effectiveness in the real world. The authors of [24] proposed a method using the well-known nonlinear least squares (NLS) and expectation-maximization (EM) algorithms to decompose human signatures into responses of body parts. A high-performance method (HAMF) to extract micro-Doppler trajectories of pedestrians using CW radar was introduced in [25]. The authors of that paper demonstrated that the proposed algorithm is able to not only better filter noise from the radar echoes but also help maintain CFAR, even in a time-varying, low-SNR environment. But like the method proposed in [24], this method requires a lot of empirical parameters to ensure the proposed algorithm works well. In [26], a 1D block based on the state-space method (SSM) was applied to extract the micro-Doppler trajectory of the human torso. Simulations verified it works well, but it should be pointed out that the trajectories of segments other than the torso are difficult to extract. The authors of [27] proposed an improved SSM (STSSM) to create a library of micro-Doppler signatures for the torso, right foot, and left foot. The experimental results showed that the peak errors of the torso trajectories and two-foot trajectories extracted by this method were 2.3% and 6%, respectively. Although such an extraction result proved that STSSM can be used as an extraction algorithm, it needs to be pointed out that when the echo component is complex, the separation result will be worse, as pointed out in [28]. Compared with the STSSM proposed in [27], no fast-time Fourier transform was included in the method proposed in [28] but an iterative process and a variable-width sliding window, which require prior knowledge. Experimental results showed that when pedestrian movement changes from simple to complex, i.e., from swinging arms to swinging arms when walking, the overall peak error exhibits an upward trend and the peak error of some joints even exceeds 10%. It should be recognized that the peak value is one of the parameters with a good response to pedestrian gait features. The peak velocity of either the torso or the foot is proportional to the pedestrian’s walking velocity. In addition, the walking cycle can be estimated by the time difference between consecutive peaks. Therefore, the more accurate the separated time–speed curve, the more reliable the extracted gait feature and the higher the classification accuracy rate.

In this paper, in order to extract robust physical gait features, we consider making some improvements to the SSM. First, we use ZCA whitening and a searching algorithm to preprocess the signal and obtain two estimated echoes representing the torso and feet. Therefore, we do not need to estimate the model order, which is crucial in the methods proposed in [27,28], whereas the model order adopted in our work is constant (1). The SSM is adopted to decompose the estimated signal of the torso and feet. On this basis, the time–velocity curve of the torso and the approximate time–velocity curve of the feet can be extracted. Furthermore, on the basis of the two extracted curves, the walking cycle, stride length, and maximum movement speed of the torso and feet can extracted for pedestrian classification. The key contributions of this paper are outlined as follows:

- Through preprocessing, not only can the torso trajectory be identified with less error, but a valuable approximate foot trajectory can also be obtained to extract features when the model order is larger.
- There is no need to estimate the model order because it can be considered constant (1) after preprocessing.
- The recognition performance is assessed based on real measurements.

The remainder of this paper is organized as follows. An introduction to the modeling of a moving human and the entire algorithmic process is given in Section 2. Section 3 presents some simulations to verify the effectiveness of the proposed algorithm. In Section 4, the data collected in experiments are used for feature extraction, and SVM is employed for walking target classification experiments to demonstrate the effectiveness of the proposed algorithm. Finally, Section 5 presents the conclusion.

## 2. Walking Human Model and Feature Extraction Algorithm

In this section, we present some preliminary knowledge and an introduction to the proposed algorithm.

### 2.1. The Moving Model of Humans

As we all know, a radar system transmits an EM wave signal to an object and receives signals back from the object. The echo reflects the EM scattering characteristics of the object, and these characteristics are very important for radar target recognition. The Doppler signature of a walking human be summarized in two parts: the frequency shift caused by the movement of the human torso and the micro-Doppler frequency side shift generated by the micro-motion of swinging limbs.

To describe human walking kinematics, the Boulic model [29], which is a mathematical parameterized model based on experimental data from biomechanics, is used. In addition to the mathematical description of the movement of the torso, the model also describes 15 other parts, including the hands, arms, feet, etc.

**Figure 1:**
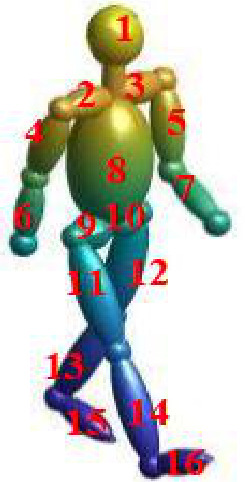
The walking model of a human.

As shown in Figure 1, the human walking echo model can be described as follows:

(1) $$s\left(t_{k}\right)=\sum_{i=1}^{16}\sigma_{i}e^{\alpha_{i}-2j\pi f\frac{2r(t_{k})}{c}},\;k=1,2,\ldots ,N,$$

where $s(t_{k})$ is an echo sequence composed of 16 echoes that come from 16 different body joints, *c* is the speed of light, $\sigma_{i}$ is the radar cross-section (RCS) of the *i*th segment, $\alpha_{i}$ denotes the decay/growth associated with the *i*th frequency, *f* is the carrier frequency, and $r(t_{k})$ is the scalar distance from the radar to the scattering point.

**Figure 2:**
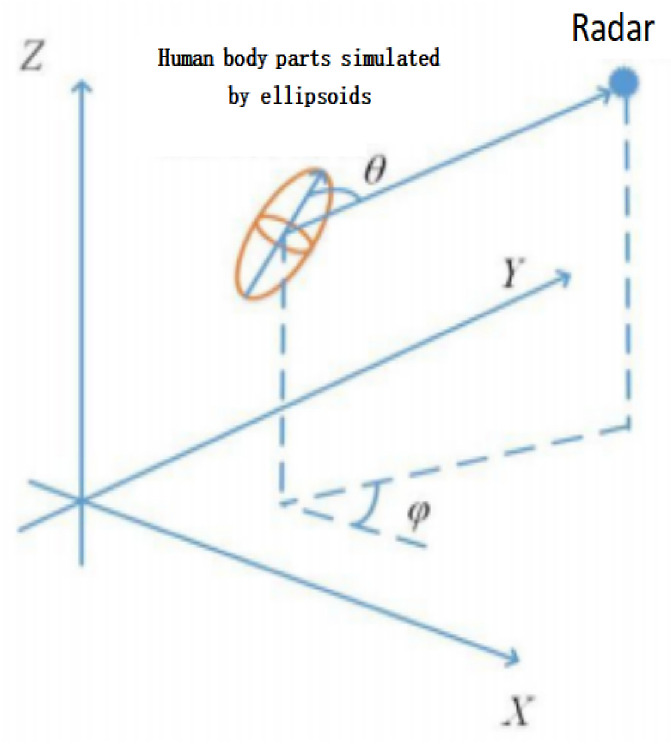
Schematic of ellipsoid RCS.

The radar cross-section (RCS) model of an ellipsoid is shown in Figure 2, where *a*, *b*, and *c* denote the lengths of the three semi-axes of the ellipsoid along the three respective coordinate axes. θ and φ represent the incident angle and the azimuth angle, respectively, which describe the orientation of the ellipsoid relative to the radar.

The RCS of each ellipsoid can be calculated using the following formula:

(2) $$RCS_{ellip}=\frac{\pi a^{2}b^{2}c^{2}}{{\left(a^{2}\sin^{2}\theta \cos^{2}\varphi +b^{2}\sin^{2}\theta \sin^{2}\varphi +c^{2}\cos^{2}\theta \right)}^{2}},$$

where

(3) $$\theta =\arctan\left(\frac{\sqrt{x^{2}+y^{2}}}{z}\right)$$

and

(4) $$\varphi =\arctan\left(\frac{y}{x}\right).$$

The RCS values are obtained by reasonably setting the dimensional parameters for each human body segment and adopting an experimental scenario with $\theta =90^{\circ }$ and $\varphi =0^{\circ }$ are presented in Table 1.

### 2.2. Feature Extraction Algorithm

When a person is walking, different segments have different frequency (or speed) distribution ranges, so a pedestrian echo can be preprocessed based on the frequency density distribution function.

ZCA whitening, as an important preprocessing algorithm, reduces input data redundancy and has been widely adopted. Kalapos and Gyires-Toth used it for feature whitening in self-supervised image learning, improving representation quality [30]. Wang et al. incorporated it into autoencoders for industrial anomaly detection, enhancing sensitivity to anomalies [31]. Inspired by these studies, we introduce ZCA whitening into our method.

The input data after whitening has the following properties: (1) The correlation between features is low, and (2) all features have the same variance. Because the energies of different segments of the human body have different statistical characteristics [32], ZCA whitening is first used to divide the human body echo into different zero-phase components.

In order to meet the ZCA whitening usage conditions, a sliding window is used to transform the 1×N single-pulse echo ($S(t_{k})$) in (1) into an M×K matrix:

(5) $$X={\left[\begin{array}{c} x_{1},x_{2},x_{3},\ldots ,x_{M} \end{array}\right]}^{T},$$

where *M* is the number of channels, K=N−M+1, and each channel is a sequence of 1×K. On this basis, ZCA whitening can be applied to process each channel. First, the average of each channel is counted, and each channel is decentralized:

(6) $$\bar{X}={\left[\begin{array}{c} x_{1}-\mu_{1},x_{2}-\mu_{2},x_{3}-\mu_{3},\ldots ,x_{M}-\mu_{M} \end{array}\right]}^{T}.$$

Second, the covariance matrix of the input matrix (*X*) is calculated:

(7) $$\Sigma =\frac{1}{M-1}\sum_{i=1}^{M}\left(x_{i}-\mu_{i}\right){\left(x_{i}-\mu_{i}\right)}^{H}.$$

Third, the eigenvalue matrix (Λ) of Σ and the corresponding eigenvector matrix (*U*) are calculated. Then, the whitening matrix (*W*) can be obtained:

(8) $$W=\Lambda^{-1/2}U.$$

Finally, after PCA whitening, the data are transformed back to the original space:

(9) Z=WX.

In (9), *Z* is a M∗K matrix, each row of which is a zero-phase component. After obtaining *M* components, we need to search for two components that can best characterize the torso and feet as the estimated signals. In our research, ZCA whitening is manually implemented using the basic functions of MATLAB R2022a without any ready-made standard functions.

The search algorithm for the torso and feet is outlined as follows:

- Step 1: First, initialize the total distance (*D*) to a very large value and calculate the frequency probability density function of each component.
  Specifically, we employ Short-Time Fourier Transform (STFT) to obtain the time-frequency representation of the signal. Let x(τ) denote the received signal and w(τ−t) be a sliding-window function (e.g., Hamming window) centered at time *t*. The time-frequency spectrum (S(t,f)) is calculated as follows:
  (10) $$S\left(t,f\right)=\int_{-\infty }^{+\infty }x\left(\tau \right)w\left(\tau -t\right)e^{-j2\pi f\tau }d\tau .$$
  To derive the frequency probability density, we normalize the spectral magnitude (or power) at each time instant (*t*). The probability of the signal energy appearing at the *m*-th frequency bin is computed as
  (11) $$f_{m}\left(t\right)=\frac{\left|S(t,f_{m})\right|}{\sum_{k=1}^{M}\left|S\left(t,f_{k}\right)\right|},$$
  where *M* is the total number of frequency bins. Consequently, the frequency probability density vector (F) at time *t* is denoted as follows:
  (12) $$F=\left[\begin{array}{cccc} f_{1} & f_{2} & \ldots & f_{M}. \end{array}\right].$$
  This normalization ensures that $\sum_{m=1}^{M}f_{m}\left(t\right)=1$.
- Step 2: Select three probability density functions in *F* as three centers and initialize a variable (Dis) to 0.
- Step 3: Take one of the remaining components and calculate the Euclidean distance between it and each of three central components:
  (13) $$\left\{\begin{array}{c} d_{ij}=∥f_{j}-f_{i}∥\\ i=1,2,\ldots ,M-3\\ j=1,2,3 \end{array}\right.,$$
  where $f_{j}$ indicates the probability density function of the *j*-th center and $f_{i}$ represents the probability density function of the *i*-th component. $d_{ij}$ represents the Euclidean distance betweentwo probability densities.
- Step 4: Take the smallest distance and count it into Dis:
  (14) $$Dis=Dis+min(d_{i1},d_{i2},d_{i3}).$$
- Step 5: Repeat Step 3 until all M−3 components have been selected. Compare Dis with the total distance (*D*). If Dis is less than *D*, update *D* and record the serial numbers of the three centers:
  (15) D=min(D,Dis).
- Step 6: Repeat Step 2 until all possible options for the center component are selected. Then, the smallest distance (*D*) and the three corresponding components are obtained.
- Step 7: Calculate the average amplitude of the three components and employ the component with the largest amplitude as the estimated torso echo, which is denoted as $\hat{s_{t}}$, and the component with the smallest amplitude as the two-foot estimated echo, which is denoted as $\hat{s_{f}}$. The average amplitude of the estimated torso echo is recorded as a feature to characterize the size of the pedestrian’s torso.

What needs to be pointed out is that after ZCA whitening, *M* zero-phase components can be obtained, but different components are not completely decorrelated. Therefore, each component is not only composed of the echo of a specific human micro-motion part. Taking into account the principle of using the state-space method to identify the time–velocity curve, a smaller order of input estimation is beneficial for feature extraction. In contrast with the traditional methods of decomposition regrouping and reconstruction, this paper does not superimpose each component but searches for two components that can best characterize the torso and feet as the estimated signals.

The state-space method (SSM) was successfully used to extract torso and foot trajectories from the fourth-order model in [27], and singular value decomposition (SVD) was used as a noise reduction algorithm in the data preprocessing stage. The noise reduction process needs to estimate the model order first. In this paper, ZCA whitening and a searching algorithm for estimated echoes are employed first, which one can also think of as a data preprocessing operation. The advantages are outlined as follows:

- There is no need to estimate the model order, which can be regarded as 1.
- The results are shielded from the influence of other segments on the extraction of torso and foot trajectories to a certain extent, so the approximate torso and foot trajectories can be obtained with less error than using the method proposed in [26], which will be shown in Section 3.

Then, for $\hat{s_{t}}$ (as well as for $\hat{s_{f}}$), the input–output relationship for the general autoregressive moving average (ARMA) can be described by the following state-space equations:

(16) $$\left\{\begin{array}{cc} x(t_{k+1}) & =Ax\left(t_{k}\right)+Bw\left(t_{k}\right)\\ y(t_{k}) & =Cx(t_{k}) \end{array}\right.,$$

where $w(t_{k})$ and $y(t_{k})$ are the input and output, respectively; *A* is the state transition matrix; and *B* and *C* are constant matrices. The transfer function (H(z)) can be obtained by incorporating the the z-transform into (16):

(17) $$H\left(z\right)=C{\left(zI-A\right)}^{-1}B,$$

where *I* is an identity matrix. We observe that the poles of the model are the eigenvalues of matrix *A*, and the zeros of the model are the eigenvalues of matrix (A−BC). Thus, according to (17), the relationship between the output ($y(t_{k})$) of the model and the state-space parameters for any positive value of *k* can be written as follows:

(18) $$y\left(t_{k}\right)=CA^{k-1}B.$$

To estimate matrix *A*, the Hankel matrix needs to be constructed first:

(19) $$H=\left[\begin{array}{cccc} x(t_{1}) & x(t_{2}) & \ldots & x(t_{l})\\ x(t_{2}) & x(t_{3}) & \ldots & x(t_{l+1})\\ \vdots & \vdots & \vdots & \vdots \\ x(t_{n-l+1}) & x(t_{n-l+2}) & \ldots & x(t_{n}) \end{array}\right],$$

where *n* is the length of a sliding window, that is, each time we select a fixed-length sequence to construct the Hankel matrix and l=[n/2], which is defined by the largest integer smaller than or equal to n/2. Then, the Hankel matrix can be further decomposed into

(20) $$H=\left[\begin{array}{cc} U_{sn} & U_{n} \end{array}\right]\left[\begin{array}{cc} \sum_{sn} & 0\\ 0 & \sum_{n} \end{array}\right]\left[\begin{array}{c} V_{sn}^{*}\\ V_{n}^{*} \end{array}\right],$$

where $\left[U_{sn}\;U_{n}\right]$ and $\left[V_{sn}\;V_{n}\right]$ are left-unitary and right-unitary matrices, respectively, and $\sum_{sn}$ and $\sum_{n}$ are diagonal matrices with singular values in the signal and noise subspace, respectively. By choosing the appropriate order to reconstruct the Hankel matrix, the corresponding number of trajectories can be estimated later. In [27], the knee-point method was adopted to determine the model order as 4, and the trajectories of the torso, left foot, and right foot were estimated accordingly. For the previously obtained $\hat{s_{t}}$ and $\hat{s_{f}}$, through the spectrogram obtained by STFT, which will be shown in Section 3, we can consider that in the two sets of estimates, the respective body-segment echoes are dominant, so we can directly set sn to 1:

(21) $$\tilde{H}=U_{1}\Sigma_{1}V_{1}.$$

Through observable and controllable decomposition, $\tilde{H}$ can be expressed as follows:

(22) $$\tilde{H}=\tilde{\Omega }\tilde{\Gamma },$$

where $\tilde{\Omega }$ and $\tilde{\Gamma }$ are observability and controllability matrices respectively. Then, the state-transition matrix *A* can be obtained as in [33]:

(23) $$A={\left({\tilde{\Omega }}_{-rl}^{*}{\tilde{\Omega }}_{-rl}\right)}^{-1}{\tilde{\Omega }}_{-rl}^{*}{\tilde{\Omega }}_{-rf},$$

where ${\tilde{\Omega }}_{-rf}$ is acquired by deleting the first row of $\tilde{\Omega }$ and ${\tilde{\Omega }}_{-rl}$ is obtained by deleting the last row of $\tilde{\Omega }$.

After calculating and recording the eigenvalues of matrix *A*, the sliding window is moved, and the steps of the SSM algorithm are repeated. Finally, a sequence of eigenvalues of length N−n+1 can be obtained. Then, the sequence of decay/growth ($\alpha_{i}$) and the instantaneous frequency trajectory can be obtained:

(24) $$\alpha_{i}=-\frac{log\left|\begin{array}{c} \lambda_{i} \end{array}\right|}{\Delta t},\;i=1,2\ldots ,N-n+1,$$

(25) $$f_{i}=-\frac{\phi_{i}}{2\pi \Delta t},\;i=1,2\ldots ,N-n+1,$$

where $\lambda_{i}$ denotes the eigenvalues; $\phi_{i}$, as appearing in (25), refers to the phase of the eigenvalue ($\lambda_{i}$); and Δt is the sampling interval.

After applying the SSM for $\hat{s_{t}}$ and $\hat{s_{f}}$, the time-frequency trajectory of the torso and feet can be obtained, respectively. Then, the classification features based on the two trajectories can be extracted: stride length, walking period, maximum torso movement speed, and maximum foot movement speed.

The processing steps of the proposed feature extraction algorithm are summarized as follows:

- Acquire raw radar data.
- Filter out static background noise for raw data.
- Use ZCA whitening on the echo to obtain *M* groups of zero-phase components.
- Regroup the *M* components into three categories and select two components as the estimation echo for the torso and feet.
- Apply the SSM to two complex sequences and extract the trajectories of the torso and feet.
- Extract features based on trajectories.

## 3. Simulation Results

The theory and methodology of the ZCA whitening and searching algorithm for estimated echoes and the SSM to extract features of human movement are discussed in the aforementioned sections. In what follows, we will demonstrate the effectiveness of the proposed method by simulations.

The Boulic model is employed to simulate the echo. There are two inputs for this model: walking velocity and height. For a given walking speed and height, the relative length of a walking cycle is empirically expressed as $R_{c}=1.346\sqrt{v_{r}}$, and the walking cycle is expressed as $T_{c}=R_{c}/v_{r}$.

**Figure 3:**
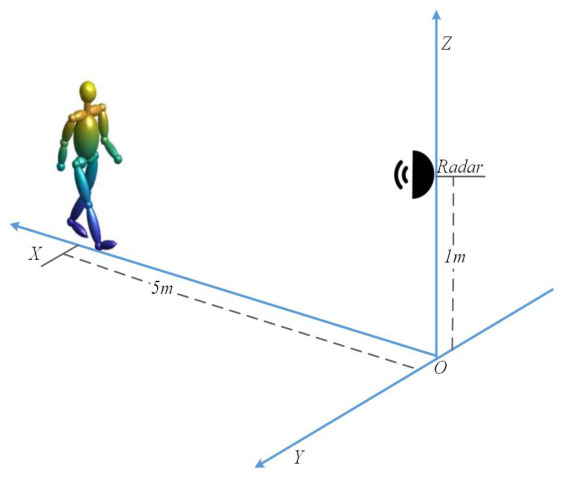
Human walking scenario.

**Figure 4:**
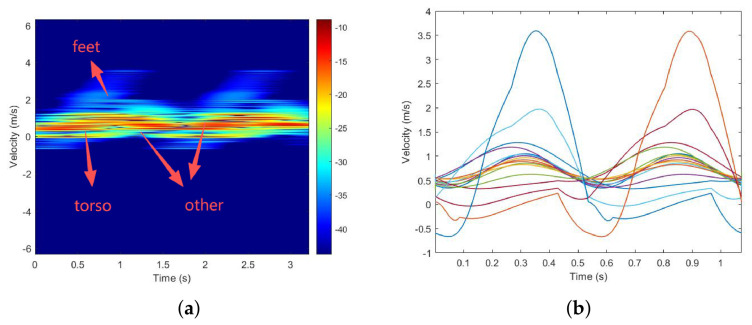
Simulated result of a walking human. (**a**) STFT result; (**b**) time-velocity curve of 16 segments.

We first locate the radar system at the original point—1 m above the ground. The object is at the LOS of the radar and is 5 m away from the radar, walking toward the radar, as shown in Figure 3. The height of the object is set to 1.70 m, and the walking velocity is set to 0.7 m/s. The carrier frequency of the radar system is 15 GHz, and the range resolution is 0.01 m. We simulate a one-period pedestrian echo sequence through the Boulic model, then perform STFT on it, the result of which is shown in Figure 4a, with the time–velocity curves of each segment shown in Figure 4b.

At the first peak in Figure 4b, the segments, represented in descending order of speed, are: left foot, left lower leg, right lower arm, left upper leg, right upper arm, left hip, right shoulder, head, torso, right foot, right lower leg, left lower arm, right upper leg, left upper arm, right hip, and left shoulder.

Based on Figure 4a, pedestrian echoes are categorized as torso, limbs, and feet, which correspond to high amplitude with low frequency, medium characteristics, and low amplitude with high frequency, respectively. In Figure 4b, for the time domain, the echoes exhibit periodicity. With the exception of the the torso, which operates at twice the gait frequency, the remaining parts share the same gait cycle. Additionally, left and right symmetric parts display a half-cycle phase difference due to their opposite swinging directions.

**Figure 5:**
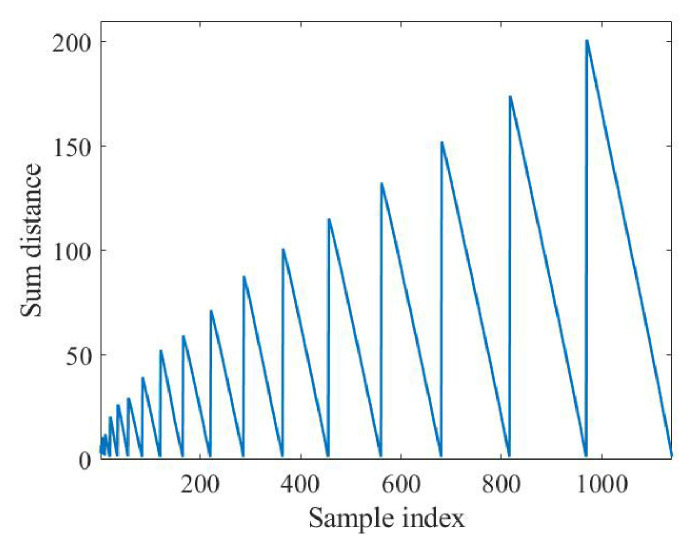
Searching of estimated echoes.

A sliding window is first used to convert the echo into an input matrix (*X*) with 20 channels. Then, ZCA whitening is applied to get 20 zero-phase components. Furthermore, the searching algorithm is employed to find two estimated echoes. The results of 1120 distance calculations are shown in Figure 5.

**Figure 6:**
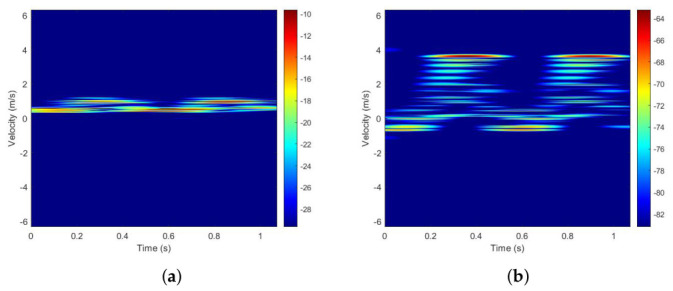
STFT result of 2 components: (**a**) component 2; (**b**) component 10.

It can be seen from Figure 5 that the distance is periodic, which means that there are some specific components. When these components are selected as the center, a smaller distance can be obtained, which explains the feasibility of the probability density as the principle of the search. Finally, when the 2nd component, the 6th component, and the 10th component are selected as the estimated echo, the distance is the smallest. According to the magnitude, the 2nd component represents the torso and the 10th component represents the feet. Short-time Fourier transform is performed on the two components of each echo component, the result of which is shown in Figure 6.

Comparing the regrouping results with those reported in [32], there is less noise caused by other segments in the spectrograms of the torso and feet in Figure 6, that is, the signals of the torso and feet are dominant in their respective spectra. On the other hand, because the employed component is not completely decorated and retains a little echo from other segments, the amplitude intensity is inevitably greater than the actual amplitude intensities of the torso and feet.

**Figure 7:**
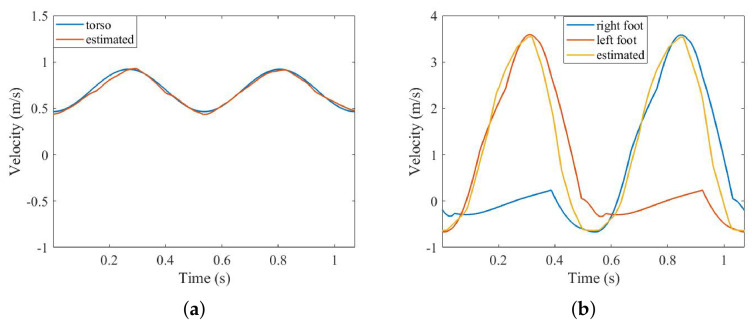
SSM result of 2 components: (**a**) torso curve; (**b**) feet curve.

The SSM is applied to extract the time–velocity curve of the torso and feet. In this process, a sliding-window length of 200 pulses turned out to be the optimum choice. The estimated time–velocity curve is shown in Figure 7.

What needs to be explained in Table 2 is that, regarding the estimation of the walking cycle, we first obtain the left and right peaks from the curve, then count all the points near the two peaks where the speed change is less than 0.2 m/s. By calculating the average times of two sets of points, their interval value can be obtained; the interval time is double the estimated value of the walking cycle.

Figure 7 shows the extracted time–velocity curve of the torso and feet, which, combined with Table 2, shows see the following: (1) The extracted torso time–frequency curve traces the original curve well on the whole. At the same time, for some characteristic values, such as the peak value and walking cycle, the error of the extracted value is less than 4%. (2) The extracted time–velocity curve for the feet does not completely follow the original curve; the values of the feet at the low velocity are lost, resulting in the extracted curve being the envelope of the curve for the feet. However, considering that when pedestrians are walking, only one foot is moving at a time, while the other is resting on the ground, the moving speed is very slow—close to 0. Therefore, the tracking effect shown in Figure 7b is acceptable [28].

In addition to the above four features, we also integrate the two time–velocity curves to estimate the stride length (because the two-foot curve contains two parts, the integral is divided by 2). The model stride value is 0.74 m, and the estimated values obtained from the time–velocity curve of the torso and feet are 0.73 m and 0.69 m, respectively, with errors of 1.35% and 6.76%, respectively. It can be seen that although the low-speed part of the time–speed curve of the feet is lost, because the low-speed part corresponds to the resident period of the corresponding foot, the motion speed of the foot is nearly 0, so the stride-length estimation error of the feet is less than 7%, which is an acceptable level.

**Figure 8:**
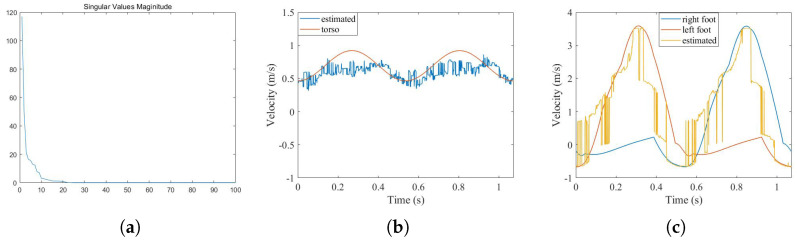
SSM result without preprocessing: (**a**) knee point of singular values; (**b**) torso curve; (**c**) feet curve.

In order to verify the effectiveness of ZCA whitening and the searching algorithm as preprocessing methods, the SSM is directly performed on the original echo to extract the corresponding features. Because there is no preprocessing now, the order of the model needs to be estimated first. The order of the model is 10, as shown in Figure 8a using the knee point method. With the adoption of the SSM, 10 groups of estimated echoes are obtained. After solving the time–frequency curves, we manually select the two curves closest to the trajectory of the torso and feet, as shown in Figure 8b,c.

Table 3 presents a comparison of the features between the extracted values and real values. One can see the following: (1) The extracted torso time–frequency curve roughly tracks the original curve, although the overall result is not as good as before. With the exception of the period of the feet, the error of other extracted feature values is also greater than the former. (2) The extracted time–velocity curve for the feet still loses low-velocity information. Furthermore, because the SSM is a method of model estimation and the echo contains more micro-movement information under a larger PRF compared with [27], the echo does not only include micro-movement information of the torso and feet but also that of the arms, legs, etc., which means that the model order is very large. In this case, the estimation of the state transition matrix (*A*) requires a large calculation, with the estimation error inevitably increasing. As a result, the overall result of the extracted time–velocity curve is poor, and the characteristic parameter errors of stride length and peak value are much larger than those of the proposed method.

**Figure 9:**
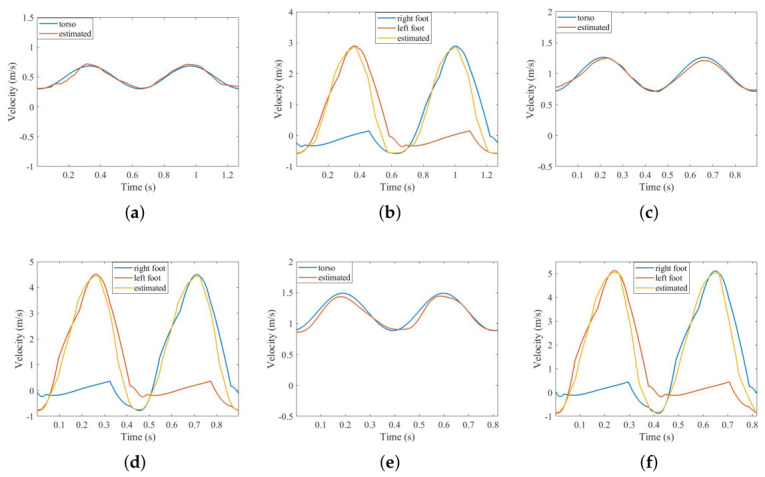
Extracted result for 3 other conditions: (**a**,**b**) 165 cm height 0.5 m/s, velocity; (**c**,**d**) 175 cm height, 1.0 m/s velocity; (**e**,**f**) 180 cm height, 1.2 m/s velocity.

Furthermore, by changing the height and walking velocity of the Boulic model, three other human walking echoes are simulated. The classification features are extracted from the simulated echoes, which are shown in Figure 9, with the corresponding results shown in Table 4.

It can be seen from Table 4 that, in terms of maximum speed estimation, the error of the two curves is less than 3%, and the maximum velocity estimation error for the feet is smaller. In addition, because the range of speed change of the torso is smaller compared with that the feet, the estimated stride length based on the torso curve is better than that based on the curve of the feet. We employ the peak method to estimate the period, and the speed range of the feet is much larger than that of the torso, that is, the peak value is more obvious than that of the torso, so the period error based on the curve of the feet is smaller. Furthermore, the amplitude error is large, but the amplitudes extracted from the four sets of simulation signals are 0.60, 0.65, 0.72, and 0.82, and the corresponding heights are 165, 170, 175, and 180, so it can be seen that the extracted amplitudes are able to reflect the size of the human body to a certain extent. However, considering that the clothing of pedestrians in different seasons and temperatures will affect the echo intensity, we only analyze the echo intensity in the previous extraction results. In the next experiment, in order to verify the effectiveness of the micro-motion feature as much as possible, the feature of echo intensity is eliminated.

The SSM depends on computationally intensive dynamic order estimation and high-dimensional singular value decomposition (SVD). In contrast to the traditional SSM, our algorithm significantly reduced complexity. We introduce ZCA whitening and frequency probability-based echo search to extract effective components. The order of the SSM model is fixed at 1; in this way, the dimension of the matrix operations is substantially reduced. While maintaining high accuracy, this study has achieved a higher speed. Furthermore, the calculation expense of preprocessing steps can be neglected.

## 4. Experimental Results

**Figure 10:**
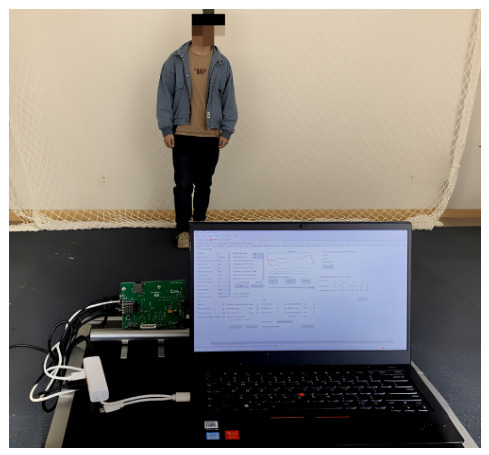
Testing scenario.

In the simulation example described earlier, the proposed algorithm was tested with the Boulic model. In this section, experimental data is utilized to validate the algorithm’s feature extraction capability. A 77 GHz FMCW radar system (IWR1642) is 5 m away from the subject and 1 m above the ground, as shown in Figure 10. The frequency sweeps from 77.0359 GHz to 80.1011 GHz. The number of A/D samples in one chirp is 256, and the A/D sampling rate is 2 Mb/s. The number of chirps in one frame is 256. The physical characteristics of the targets are given in Table 5. The data obtained by FMCW radar can be formulated as a range-Doppler matrix.

As a classic short-range radar system with the merits of high Doppler resolution and low power consumption, FMCW radar is well-suited for the detection of human gait [34]. Considering that the location of the subject is known in the experiment, we collapsed the range dimension of the data to get a data formation of one dimension.

Even with the reduced range dimension, the preserved Doppler information is still sufficient to achieve effective separation and accurate feature extraction using the proposed method, which demonstrates the practicality and robustness of FMCW radar in gait measurement applications.

**Figure 11:**
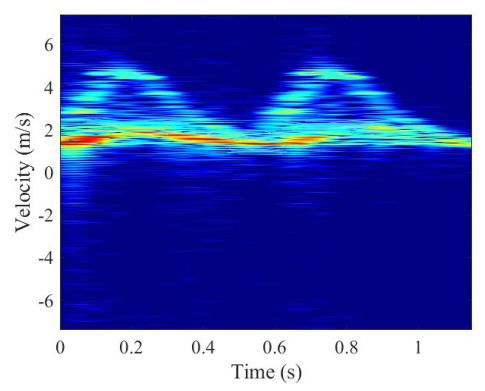
STFT result of the collected data.

One walking cycle is collected each time, and each target is collected 100 times. One sample is selected to perform a short-time Fourier transform as an example, the result of which is shown in Figure 11.

It can be seen from Figure 11 that the spectrogram has two types of obvious echo modulation: the torso with relatively low velocity modulation but high echo intensity and the feet with relatively low modulation but low echo intensity. Compared with the Boulic model, some other body parts, such as hands, lower arms, shoulders, etc., are not clearly reflected in the spectrogram because of the weak echo intensity.

**Figure 12:**
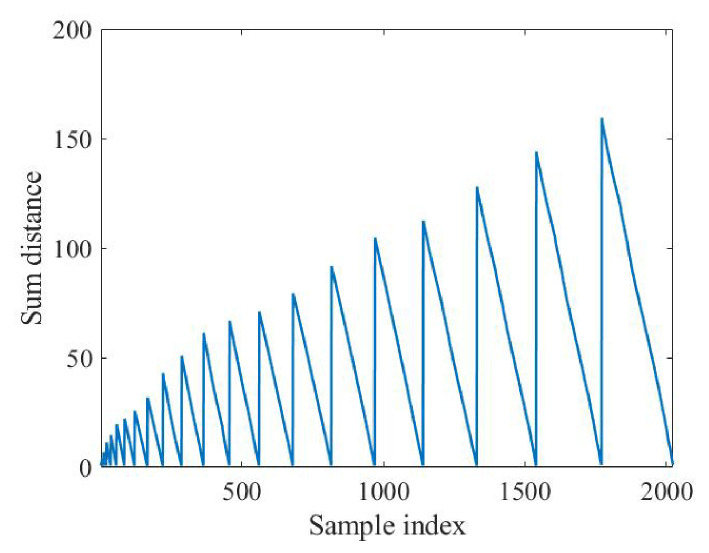
Searching of estimated echoes.

**Figure 13:**
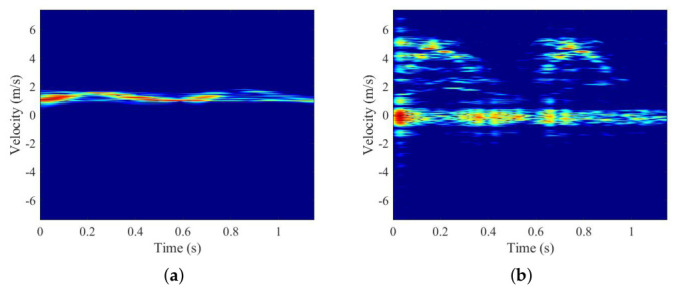
STFT result of 2 component (**a**) Component 2 (**b**) Component 6.

To extract gait features, the echo signal is processed by a sliding window to obtain a 24-channel two-dimensional signal (*X*). Then, ZCA and the searching algorithm are applied first, with the result of the distance calculation shown in Figure 12. In this experiment, component 2 and component 6 are selected. Figure 13 gives the STFT results of these two components.

**Figure 14:**
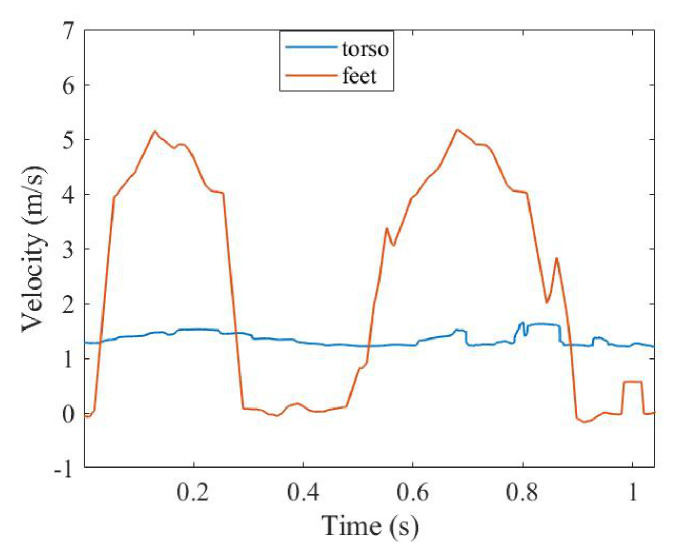
SSM result of the collected data.

The SSM is applied to two components to solve the corresponding time–velocity curve, the results of which are shown in Figure 14.

It can be seen from Figure 14 that, in contrast with the simulation, the torso time–velocity curve distribution in the experiment is not exactly a sine curve. Under such a distribution, the walking cycle calculated by the torso peaks will have a large error. On the other hand, it can be seen that the torso time–velocity curve is more concentrated, so the stride-length error estimated by the torso time–velocity curve is smaller than the error estimated by the time–velocity curve of the feet. In addition, the peaks of the time–velocity curve of pedestrians are not very obvious compared with the peak of the Boulic model. However, in walking-cycle estimation, all points near the maximum value of 0.3 m/s are counted, so to a certain extent, the lack of obvious peaks is made up for.

**Figure 15:**
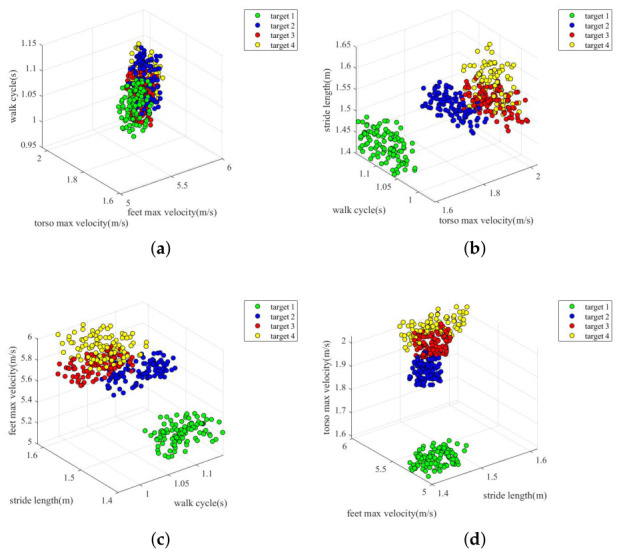
Three-dimensional distribution of gait features. (**a**) Distribution of torso velocity, foot velocity and walk cycle; (**b**) Distribution of walk cycle, torso velocity and stride length; (**c**) Distribution of walk cycle, stride length and foot velocity; (**d**) Distribution of stride length, foot velocity and torso velocity.

For easier analysis, Figure 15 shows the three-dimensional distribution diagram of the four gait features extracted from the dataset. It should be pointed out that each time the pedestrian echo is collected, the speed and cycle of the pedestrian are not fixed but fluctuate within a range. From Figure 15a, it can be seen that the walking cycle is messy, and the visibility of discrimination is not very high. In addition, at the maximum torso velocity and stride length, as can be seen in Figure 15b,c, the discrimination of target 1 is better, while target 2, target 3, and target 4 have a higher degree of overlap in the distribution range. In addition, it can be seen from Figure 15d that the discrimination degree of maximum foot velocity is the best among the four features.

**Figure 16:**
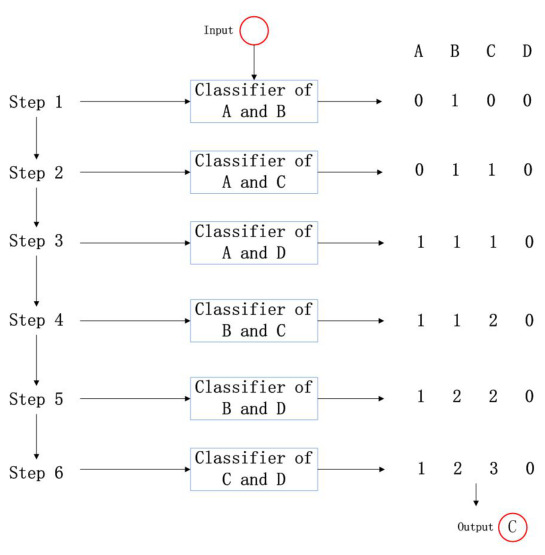
Classification process.

In order to further test the effectiveness of the extracted features, an actual classification experiment is required. Before classification, the classifier needs to be designed first. The task requirement is to classify four targets, which is a typical multi-classification scenario. Common solutions are One-vs-One (OvO), One-vs-Many (OvM), and Many-vs-Many (MvM). Considering that there are 100 samples collected from each target, in order to avoid a decrease in classification accuracy caused by sample imbalance, OvO is applied to design the classifier here. In other words, to design a classifier for every two targets, a total of six classifiers need to be trained. For each classification, the target to be classified needs to be judged bythe six classifiers in turn, and each time, the category that is determined as the result receives one point. Finally, the category with the highest score is regarded as the category of the target to be classified. The classification process is shown in Figure 16.

**Figure 17:**
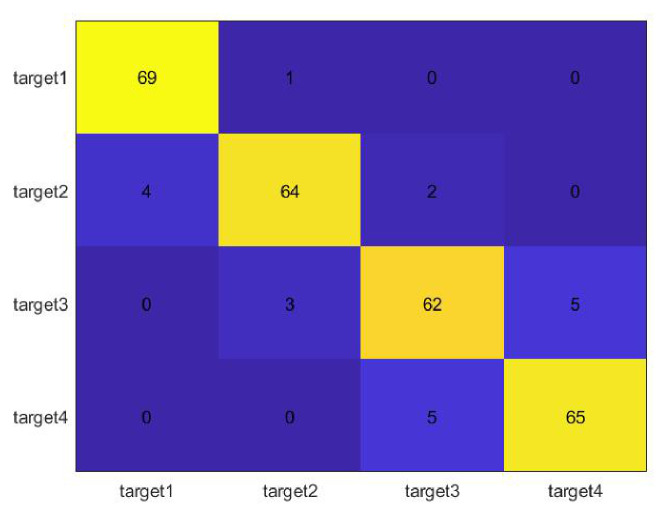
Classification result.

A support vector machine is a kind of generalized linear classifier that classifies binary data in a supervised learning manner. Its decision boundary is the maximum margin hyperplane that is solved for learning samples. SVM uses the hinge loss function to calculate the empirical risk and adds a regularization term to the solution system to optimize the structural risk. It is a classifier with sparsity and robustness. SVM can perform non-linear classification through the kernel method. It is one of the common kernel learning methods and is widely used in pattern recognition problems. We randomly select 30 samples of two target datasets each time as the training set to train one SVM, and a total of six SVMs are trained. The remaining 70 samples of each dataset are used as the testing set, and the experimental results are shown in Figure 17.

**Figure 18:**
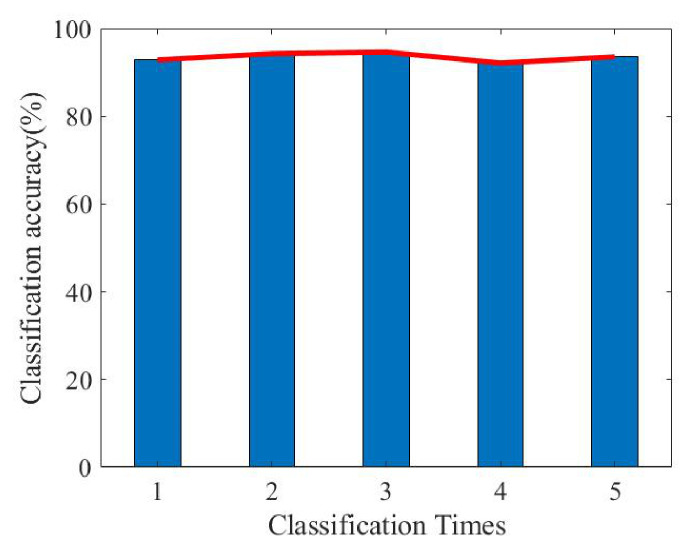
Five classification results.

The results presented in Figure 17 are consistent with the previous feature analysis. Because the gait feature of Target 1 is obviously different from the other three, the highest classification accuracy rate is reached, while the cycle and stride lengths of Target 2 and Target 3 are relatively close, so the correct rate is relatively low. The final correct rate of the four classification targets is 92.86%. We continue to randomly select 30 groups from the dataset of each target as the training set and repeat the classification experiment five times. The correct rate situation is shown in Figure 18.

It can be seen from Figure 18 that after feature extraction via the proposed algorithm, the accuracy rate of five classifications is above 90%, and the average accuracy rate is 93.5%, indicating that the proposed algorithm achieves a high classification accuracy rate in the case of a small dataset.

In our experiments, the radar was mounted at a 1 m height, and subjects walked directly toward it. To address whether our method remains robust when the radar is lowered or placed at an oblique angle, we carried out an extended analysis based on the experimental findings reported in [35]. That work specifically looked at how well mm-wave radar holds up under non-ideal installation scenarios, which closely relates to our study.

Zhao et al. utilized a 79 GHz MIMO FMCW imaging radar for human motion and posture recognition, which is similar to the 77 GHz radar employed in this work. The radar was mounted at a height of 0.75 m, and subjects were tested at five different angles ranging from 0° to 180°. According to their experimental results and the design of our algorithm, the recognition performance remains robust for non-lateral angles (0–45° and 135–180°). However, when the observation angle approaches 90°, the accuracy decreases significantly [35]. The main reason is that micro-Doppler features cannot be effectively projected along the radar line of sight. Therefore, for extreme lateral observation scenarios, the clustering logic needs to be further optimized.

It should be emphasized that we used 15 GHz for simulations, simplifying electromagnetic modeling and efficiently generating body echoes while still bringing out the intrinsic micro-Doppler differences. For experiments, we chose 77 GHz because it is widely available and has a short wavelength. A higher frequency produces larger Doppler shifts, which amplify subtle gait signatures and improve feature extraction accuracy. The algorithm does not depend on absolute frequency shifts or absolute RCS values, though. It works off relative feature patterns and geometric RCS ratios. Therefore, the same method applies consistently to both the 15 GHz simulations and the 77 GHz measurements. In future work, we will extend this to other bands—24 GHz and 120 GHz, for example—to test its versatility.

## 5. Conclusions

In this paper, an improved SSM algorithm is proposed for gait feature extraction. By introducing ZCA whitening and a searching algorithm, the order of the state transition matrix (A) in SSM is reduced, and the classification of the features of a walking human with physical meaning are extracted when the echo is complex (the return signal not only contains torso and feet echoes but also includes echoes of the head, shoulders, arms, and other body parts). Compared with the features extracted by the unimproved SSM algorithm, the error of the features extracted by the proposed algorithm is smaller. Simulation proved the effectiveness of the feature extraction algorithm. Then, compared with the torso time–speed curve, because the curve of the feet has a larger speed distribution range, the stride error estimated by it will be greater than the error estimated by the torso curve. However, conversely, in terms of cycle estimation, the error estimated by the curve of the feet is smaller. Furthermore, through a 77 GHz FMCW radar system, four pedestrian echo datasets were produced, containing 100 samples. Then, in order to test the effectiveness of the remaining features, a classification experiment was carried out. Considering that the amplitude feature is easily affected by external influences (e.g., season and temperature), it was eliminated in the actual experiment. Based on OvO, one SVM was trained for every two targets, and the final trained classifier contained six SVMs. High-level recognition performance was achieved in the experiment, and the average correct rate reached 93.5%.

Limited by the low echo intensityof some parts of the human body and the range resolution of the radar system, there are few parts of the human body that produce sufficient frequency modulation for actual data compared to simulation data. Therefore, in the future, the algorithm will be tested with a radar system with higher performance. In addition, in order to further verify the effectiveness of the algorithm, more datasets will be produced. Besides expanding the dataset, testing algorithms in an experimental environment with fewer restrictions is another aspect of future work.

We recommend selecting the cluster number in accordance with the physical characteristics of the target object. The selection of the sliding window length should be optimized to adapt to scene factors such as noise, viewing angle, and moving speed [27].

Our method has some limitations when it comes to tracking foot movement. The radar struggles to pick up weak signals from the foot when it is planted on the ground (the stance phase). As a result, our data mainly captures fast swinging motion, missing the slower parts of the step. This means we cannot calculate metrics that rely on timing, like the stance-to-swing ratio, or see exactly when the foot touches or leaves the ground. However, this does not affect the main goals of our study. We can still accurately measure stride length, walking cycles, and maximum speeds. These core features remain reliable, making our method effective for classifying gait patterns, even with limited data.

We recognize that the current experiment includes only four subjects, which limits the assessment of generalization. However, several aspects of our method support its scalability.

First, ZCA whitening normalizes feature variance, making the method less sensitive to changes in walking speed. Second, the frequency probability-based echo search does not rely on predefined motion patterns, so it can handle different gait types without retraining. Third, the extracted physical parameters—such as stride length and peak foot speed—are grounded in kinematics and should remain informative across individuals of different ages, heights, or walking styles. These features may also capture micro-Doppler modulations during turning, though this remains to be tested.

In future work, we plan to upgrade to radar systems with higher range resolution and sensitivity. This will help us capture weak echoes and micro-speed variations from the feet during the stance phase, ensuring we get complete motion data right from the source. We also intend to refine our signal processing. By incorporating human gait kinematics into our state-space method, we can better handle low-speed signals and fill in the gaps for missing trajectory segments. Finally, we will significantly expand our dataset. We aim to include at least 10 subjects with varying heights, speeds, and gait patterns to verify that our algorithm works across a wider range of real-world scenarios.

## Figures and Tables

**Table 1:** Radar cross-section (RCS) values of 16 human body segments approximated by 3D ellipsoids under normal incidence ($\theta =90^{\circ }$, $\varphi =0^{\circ }$).

No.	Body Segment	Semi-Axis *a* (m)	Semi-Axis *b* (m)	Semi-Axis *c* (m)	RCS σ (m^2^)	RCS σ (dBsm)
1	Head	0.15	0.15	0.20	0.1257	−9.0
2	Right Shoulder	0.08	0.08	0.10	0.0314	−15.0
3	Left Shoulder	0.08	0.08	0.10	0.0314	−15.0
4	Right Upper Arm	0.06	0.06	0.30	0.2827	−5.5
5	Left Upper Arm	0.06	0.06	0.30	0.2827	−5.5
6	Right Lower Arm	0.05	0.05	0.25	0.1963	−7.1
7	Left Lower Arm	0.05	0.05	0.25	0.1963	−7.1
8	Torso	0.40	0.25	0.50	0.3068	−5.1
9	Right Hip	0.10	0.10	0.12	0.0452	−13.4
10	Left Hip	0.10	0.10	0.12	0.0452	−13.4
11	Right Upper Leg	0.20	0.08	0.40	0.0804	−10.9
12	Left Upper Leg	0.20	0.08	0.40	0.0804	−10.9
13	Left Lower Leg	0.15	0.06	0.35	0.0616	−12.1
14	Right Lower Leg	0.15	0.06	0.35	0.0616	−12.1
15	Left Foot	0.10	0.05	0.15	0.0177	−17.5
16	Right Foot	0.10	0.05	0.15	0.0177	−17.5

**Table 2:** Features of a walking subject with a height of 1.70 m and a speed of 0.7 m/s (from the Boulic model and estimated features).

**Feature**	Torso amplitude	Torso (feet) cycle
**Real value**	0.42	1.07 s
**Estimated value**	0.65	1.03 s (1.07 s)
**Error (%)**	54.76	3.74 (<0.01)
**Feature**	Max velocity of torso	Max velocity of feet
**Real value**	0.92 m/s	3.59 m/s
**Estimated value**	0.93 m/s	3.54 m/s
**Error (%)**	1.09	1.39

**Table 3:** Gait feature extraction results of the traditional SSM (height: 1.70 m; walking speed: 0.7 m/s).

Index	Torso Amplitude	Torso Period (s)	Foot Period (s)	Torso Max Speed (m/s)	Foot Max Speed (m/s)	Torso Stride Length (m)	Foot Stride Length (m)
Ground Truth	0.55	1.07	1.07	0.92	3.59	0.71	0.71
Estimated Value	0.84	1.44	1.07	0.86	3.53	0.40	0.59
Relative Error (%)	52.73	34.58	0.68	6.52	1.67	43.66	16.90

**Table 4:** Average relative errors of gait features extracted by the proposed SSM.

Index	Torso Amplitude	Torso Period (s)	Foot Period (s)	Torso Max Speed (m/s)	Foot Max Speed (m/s)	Torso Stride Length (m)	Foot Stride Length (m)
Average Relative Error (%)	47.85	3.77	0.01	2.42	1.42	2.74	6.97

**Table 5:** The physical characteristics of 4 targets.

Parameter	Height	Velocity	Walking Cycle
**Value**	1.62 m	1.34 m/s	1.14 s
**Value**	1.73 m	1.45 m/s	1.10 s
**Value**	1.74 m	1.51 m/s	1.06 s
**Value**	1.76 m	1.58 m/s	1.01 s

## Data Availability

The datasets generated and analyzed during the current study are available from the corresponding author upon reasonable request.
